# Quality of Life in Children Following Treatment for a Malignant Primary Bone Tumour Around the Knee

**DOI:** 10.1080/13577149778461

**Published:** 1997-03

**Authors:** Christine Eiser, Paul Cool, Robert J. Grimer, Simon R. Carter, Imogen M. Cotter, Ann J. Ellis, Sheryl Kopel

**Affiliations:** 1 CRC Child and Family Research Group Department of Psychology University of Exeter Devon Exeter EX4 4QG UK; 2 Royal Orthopaedic Hospital Birmingham UK

## Abstract

*Purpose*. We report on the quality of life following treatment for a malignant primary bone tumour around the knee in skeletally immature children.

*Patients*. Patients (*n* = 41; mean age = 18 years; range 8–28) had all experienced chemotherapy in a neo-adjuvant setting, surgical excision of the tumour and endoprosthetic replacement.

*Methods*. Interviews were conducted separately with the child and mother and focused on mobility, body image and the impact of treatment on schooling, employment and plans for the future.

*Results*. Mobility in the group was variable. Only 12% reported that they could run with any confidence. The proportion who were able to swim (49%) or ride a bike (46%) was higher. All had experienced major disruption in schooling (mean absence following diagnosis = 12 months). Eight had repeated a school year and 41% patients reported that their schoolwork was affected. As a result of their experience, eight (six females and two males) chose health-related employment. Concerns for the future were highest among males and those with manual jobs. Three patients were receiving psychiatric support, in relation to extreme concern about the risk of recurrence. All expressed satisfaction with treatment, and older patients believed that the prosthesis gave a better quality of life than amputation.

*Discussion*. Our data suggest that outcome following limb-salvage surgery is variable. Education is disrupted. Even so, only two left school with no qualifications. Employment is most restricted among males with few qualifications who may benefit from sensitive vocational counselling.


*Sarcoma* (1997) 1, 39–45

ORIGINAL ARTICLE

### Quality of life in children following treatment for a malignant primary bone tumour around the knee

CHRISTINE EISER,[1] PAUL COOL,[2] ROBERT J. GRIMER,[2] SIMON R. CARTER,[2] IMOGEN M. COTTER,[1] ANN J. ELLIS[1] & SHERYL KOPEL[1]

[1]*CRC Child and Family Research Group, University of Exeter, Devon &* [2]*Royal Orthopaedic Hospital, Birmingham, UK*

**Abstract**

*Purpose.* We report on the quality of life following treatment for a malignant primary bone tumour around the knee in skeletally immature children.

*Patients.* Patients ($n = 41$; mean age = 18 years; range 8–28) had all experienced chemotherapy in a neo-adjuvant setting, surgical excision of the tumour and endoprosthetic replacement.

*Methods.* Interviews were conducted separately with the child and mother and focused on mobility, body image and the impact of treatment on schooling, employment and plans for the future.

*Results.* Mobility in the group was variable. Only 12% reported that they could run with any confidence. The proportion who were able to swim (49%) or ride a bike (46%) was higher. All had experienced major disruption in schooling (mean absence following diagnosis = 12 months). Eight had repeated a school year and 41% patients reported that their schoolwork was affected. As a result of their experience, eight (six females and two males) chose health-related employment. Concerns for the future were highest among males and those with manual jobs. Three patients were receiving psychiatric support, in relation to extreme concern about the risk of recurrence. All expressed satisfaction with treatment, and older patients believed that the prosthesis gave a better quality of life than amputation.

*Discussion.* Our data suggest that outcome following limb-salvage surgery is variable. Education is disrupted. Even so, only two left school with no qualifications. Employment is most restricted among males with few qualifications who may benefit from sensitive vocational counselling.

**Key words**: *bone tumour, knee, children, limb-salvage surgery, quality of life.*

### Introduction

The incidence of bone tumours (osteosarcoma and Ewing's tumour) in children is small. Prognosis has improved in recent years[1] and consequently it is now timely to consider the unique experiences and difficulties experienced by these patients. Treatment involves neo-adjuvant chemotherapy according to the relevant treatment protocol and the surgical excision of the tumour. Due to problems of limb-salvage surgery in children, some centres advocate primary amputation for children who present with a malignant primary bone tumour.[2]

Options described for limb salvage in children include endoprosthetic replacement, allografting[3] and van Nes rotationplasty.[4–6] Our centre has performed limb-salvage surgery for skeletally immature children with malignant primary bone tumours of the lower extremity since 1976.

An extensible endoprosthetic replacement consists of a massive replacement of bone that can be lengthened, a constrained knee joint and a sliding component.[7,8] The sliding component is situated in the distal femur in proximal tibial replacements and in the proximal tibia in distal femoral replacements. It consists of an intramedullary stem that crosses the physis perpendicularly and is designed to allow growth to continue at that physis.

At the time of diagnosis the expected growth is calculated according to data provided by Tupman.[9] If the expected growth exceeds 4 cm (bone age less than 14 years in boys or 12 years in girls), an extensible endoprosthetic replacement is contemplated. Otherwise, a non-extensible endoprosthetic replacement with a sliding component is inserted.[7,8]

The surgical procedure involves *en bloc* resection of the tumour and replacement. The children who have had an extensible endoprosthetic replacement

Correspondence to: C. Eiser, CRC Child and Family Research Group, Department of Psychology, University of Exeter, Exeter, Devon EX4 4QG, UK. Tel: +44 1392 264626; Fax: +44 1392 264623; E-mail: c.eiser@exeter.ac.uk.

1357-714X/97/010039-07 ©1997 Journals Oxford Ltd require regular hospital admissions in order to lengthen the prosthesis. On average, two lengthening operations per year from time of diagnosis to skeletal maturity are required in order to maintain limb length equality.[10] Limb-salvage treatment appears to offer the possibility of better psychological functioning, and intact body image. At the same time, there appears to be no difference in terms of overall survival between limb salvage and amputation.

Nevertheless, there are some disadvantages with limb-salvage surgery compared with amputation. Additional hospitalizations may be necessary depending on specific complications, including infections or loosening of the prosthesis. Care must be taken that the limb is not damaged, and children are advised not to participate in contact sports. Salvaged limbs vary in their degree of function and there can be some discomfort. Fear of injuring the effected limb, failure to reintegrate successfully with peers and reduced sporting prowess may mean that at least some children adopt a restricted life-style. Repeated school absence may also compromise school achievement. Given the extent of medical treatment involved, and the known potential complications, it is necessary to determine how far these procedures compromise children's quality of life, both in terms of physical and psychological function.

Our study is therefore concerned with the implications for quality of life of endoprosthetic replacement for a malignant primary bone tumour around the knee in skeletally immature children. A small number of studies have compared adult patients treated by amputation or limb surgery in terms of psychological outcome.[11,12] These have included patients across a wide age range and have not focused on the specific difficulties of young people. Although Sammallahti *et al.*[13] concluded that there was no increased incidence of psychiatric problems in children with osteosarcoma, all those included in this study had been treated by amputation. There has, therefore, been no study to date which has specifically addressed the impact of limb-salvage surgery on quality of life in children and young adults.

There is currently no absolute agreement regarding the measurement of quality of life in children,[14] and no agreement about the domains or areas of function which contribute to quality of life.[15] We therefore developed an appropriate interview schedule, to cover the main areas which have most frequently been cited as relevant.[14,16] In this paper, we report: (i) school and educational achievements; (ii) current employment status; (iii) physical functioning: ability to get around independently, participation in sports; and (iv) pain and body image. For patients over 18 years, general well-being was assessed using the SF-36.[17] In addition, we asked whether or not the patient was receiving psychiatric or psychological counselling. As is common following amputation or surgical procedures of this kind, physical functioning was assessed using the Functional Evaluation Index.[18]

In recognition of the increasingly aggressive nature of modern medicine, particularly in the treatment of rare or potentially life-threatening disorders, there has been a burgeoning interest in 'quality of life' work with children who have been treated for a range of diseases. While this work is believed important to determine progress in the field, it is not known how far families find it distressing to be asked to participate in such studies. In order to address this question, participants were asked to rate how distressing they had found the interview.

### Patients and methods

Approval to conduct the study was sought and obtained from the appropriate Ethics Committee. All patients who had an extensible endoprosthetic replacement of the distal femur or proximal tibia at the Royal Orthopaedic Hospital, Birmingham since 1976 were traced through medical records. For inclusion in the study, we required that patients were at least 7 years of age and had a minimal follow-up of 6 months. Patients who were experiencing metastatic disease and undergoing active treatment were excluded. Sixty-three patients were initially identified. Of the 63 patients identified through medical records, six were not contacted either because they had since been treated by amputation or were in relapse. Nine could not be contacted; they did not answer at least two letters to the last known address. One family was excluded because the parents did not speak English. A further three declined to participate. (One of these had previously refused lengthenings treatment and had not been followed up for 4 years and two mothers anticipated that they would be too distressed.)

Forty-four families were traced and agreed to take part in the study (an overall response rate of 70%). In 38 families, both child and mother were interviewed. In three families the patients took part but not their mothers, and in a further three families the mothers agreed to be interviewed themselves but did not wish to involve their child. (This included one 11-year-old who was thought to be too young by his mother and two older males, both of whom were working full-time and were 'too busy'.)

In this paper, we report the results of the 41 interviews with patients. The mean age of the group was 18 years (range 8–28), and included 24 males and 17 females. Five had been treated for Ewing's sarcoma. For the total group, 35 had received an extensible endoprosthetic prosthesis and nine a non-extensible prosthesis as previously described.

Additional demographic and medical data describing the sample are shown in Table 1.

**Table 1.** *Clinical and demographic data*

| | Not interviewed ($n = 22$) | Interviewed ($n = 41$) |
|---|---|---|
| Chronological age | 17.7 (8–25) | 18.6 (9–28) |
| Years since diagnosis | 6.8 (2–13) | 7.8 (2–15) |
| Age at diagnosis | 10.9 (7–15) | 10.8 (5–14) |
| Number of operations | 9.6 (1–19) | 9.8 (2–17) |

Patients were initially approached by letter, outlining the purpose of the study. For patients below 14 years of age, the letter was sent directly to parents. For those over 14 years, separate letters were sent to patient and parent. For all families, participation of both patient and parent was requested. However, the letter suggested that it was also acceptable for only one member of the family to participate if that was preferred. Included with each introductory letter was a reply slip, for families to return and suggest a convenient time for the interview to take place. All but three interviews were conducted in family homes. In these three cases, patients felt it would be more convenient to be interviewed at the hospital on a routine follow-up visit. Mothers and patients were always interviewed separately.

### Measures

An open-ended interview was conducted which enabled survivors to expand on their own experiences and raise particular issues which they considered unique to themselves. The interview focused on a number of themes: recall of the diagnosis, understanding of the cause, rationale for treatment, possibility of complications and need for future treatment, perception of how well informed they had been, and their own opinion about how and how much children should be told about the disease. (These data will be reported separately.)

*(i) Impact on schooling.* Estimation of length of time absent from school, both following diagnosis and for subsequent lengthenings, examinations passed, any course still being studied, whether or not patients had repeated a school year or needed special help and self-ratings of learning difficulties.

*(ii) Work.* Employment status, work satisfaction, impact of illness on choice of work.

*(iii) Mobility.* Competence to take part in different sporting activities, ability to run, ride a bike, swim, walk upstairs, walk downstairs, drive a car (manual or automatic), difficulties experienced travelling.

*(iv) Pain and body image.* Patients were asked if they experienced any pain at all or only after strenuous activity. They were also asked to report if they used analgesics and how frequently. With regard to body image, patients were asked their views about the appearance of their leg and whether or not they dressed to disguise it.

*(v) Emotional well-being.* We recorded whether or not the patient was receiving, or had ever received, psychiatric help or psychological counselling. In addition, patients above 18 years completed the SF-36.[17] This measures generic health and includes eight multi-item scales containing between two and ten items each, and a single item measure of reported health transition. The subscales are designed to measure: physical functioning (extent to which health limits physical activities such as self-care, walking, climbing stairs); role physical functioning (extent to which physical health interferes with work or daily activities); bodily pain (intensity of pain and effect of pain on normal work); general health (personal evaluation of health); vitality (feeling energetic compared with feeling tired and worn out); social functioning (extent to which physical health or emotional problems interfere with normal social activities); role functioning emotional (extent to which emotional problems interfere with work or daily activities); mental health (general mental health, including depression, anxiety); and reported health transition (evaluation of health compared with 1 year ago). This scale is currently being used widely and was chosen because of the availability of norms for the British population.[17]

*(vi) Functional evaluation of reconstructive procedures.*[18] This scale is used as a standard assessment of function following surgical treatment for bone tumours. It includes ratings of six categories: pain (none-severe); function (no restriction–total disability); and emotional acceptance of the limb (enthusiastic–dislikes); use of supports (none–crutches); limitations in walking (unlimited–unable without aid); and gait (normal to major handicap). Ratings are made on a 0–5 scale and scores summed to give an overall rating. A numerical score and percentage rating are calculated, with higher scores indicating better functioning and emotional acceptance. This scale was completed by patients only.

*Treatment of data.* Standardized measures were scored as directed in the appropriate publications. Interviews were transcribed and later coded by two authors independently. Inter-rater reliability was initially good (92%) and disagreements resolved by discussion. Categorical data were summarized as percentages. Student *t*-tests were used to compare mean scores on the SF-36 with population norms and to compare ratings of function made by patients with those made previously by surgeons. Significance was accepted at the $p < 0.05$ level.

### Results

Comparisons were first made between those with Ewing's sarcoma and the rest of the sample. No differences were identified in terms of Enneking scores[18] nor on any of the psychological measures. Despite the increased toxicity of treatment for those with Ewing's sarcoma, the groups did not differ in self-estimates of learning difficulties, time away from school, or whether or not they had repeated a school year. In all remaining analyses, therefore, the groups were combined.

*(i) School and educational achievements.* The majority of children were attending or had attended regular state schools ($n = 38$). Two attended private school and one had attended a school for the physically handicapped. Immediately following the diagnosis, all children had missed a considerable time from school (median = 12 months, range = 6–15 months). In addition, children with an extensible prosthesis experienced further frequent absences associated with each lengthening. On each occasion, the average time away from school was 5 days (range = 2–7 days). The most common reason for these absences (beyond the hospitalization period) was associated with practical difficulties of returning to school (fear of being knocked over by crowds, inability to use stairs).

Eight had repeated or were currently repeating a school year. A further three children were having extra teaching help in school on a regular basis. This included one patient (male) who had dyslexia problems which pre-dated the diagnosis. Two females reported learning difficulties which they felt were associated with treatment. In practice, they described difficulties in concentration and understanding instructions. (There were no patients with Ewing's sarcoma in these groups.)

Among the group, six were too young to have attempted any public examinations. Among the remainder, 12 (29%) were taking or had completed education to GCSE standard (or the equivalent Intercert awarded in Ireland). Seven (17%) were taking vocational courses (e.g. hotel catering). Two left school with 'A' levels and nine (22%) were registered or had completed a course leading to a degree, and three (7.3%) were taking or had completed higher degrees. Only two (5%) had left school with no qualification and had no plans to change this.

Twenty-four (59%) reported that the illness or its experience had no effect on their choice of subjects studied. However, 18 (41%) felt that their choices had been reduced because of lost schooling. (French and maths were most frequently cited as subjects where difficulties were experienced making up for lost time.)

*(ii) Current employment status.* Twenty-six (63%) were still in full-time education. Among the rest, three (7%) were unemployed, 11 (27%) were in full-time employment and one was a housewife and mother. Four reported that their work was too demanding for them and six thought it was 'sometimes'. One female was wanting to give up her clerical post in order to return to full-time education.

All patients were asked whether they thought the illness had made or would make any difference to their choice of work. We were able to identify four groups:

(a) *Eight patients did not make appropriate responses.* All were among the youngest in the sample and had no idea what they would do or were very unrealistic (wanted to be a pop star!).
(b) *Ten reported that the illness made no difference to their choice of work or their perceived opportunities at work.* This included two at university, both of whom anticipated taking sedentary jobs which would not be effected by their condition. Others in this group in fact were unemployed or had low paid jobs and were unskilled. However, they pointed to their friends and siblings in similar positions and did not expect that their lives would have been different in other circumstances.
(c) *The largest group* (n = 14) *felt that their opportunities were limited as a result of the illness.* All reported that restricted mobility was a limitation on job opportunities. This group included four women, all of whom had left school with no or very few qualifications. Among the men, although the majority had few qualifications, one had a degree (in engineering).
(d) *A final group* (n = 8) *reported that their experiences had directed their choice of work.* All were employed in social or health environments; all but two were female. (The two males were also employed in the Health Service. Although they conceded their experiences might have made some difference to their choice of work, they were definite that they were inclined to pursue these careers anyway.)

Only one male (and no females) anticipated that there might be work problems in the future. Although currently employed in a skilled manual job, he recognized that it would not be possible to continue indefinitely and was planning to take a business course. (In addition, it is important to note that from the separate interviews involving mothers, we learned that the two males who were 'too busy' to be interviewed were in full-time manual employment.)

**Table 2.** *Participation in sports and physical activities (%)*

| | Bike | Swim | Run | Upstairs | Downstairs | Drive |
|---|---|---|---|---|---|---|
| Too young | — | — | — | — | — | 42 |
| Unable | 22 | 7 | 56 | 0 | 2 | 5 |
| Some difficulty | 32 | 34 | 30 | 68 | 68 | 2(auto.) |
| No difficulty | 46 | 49 | 12 | 30 | 30 | 51 |

*(iii) Physical functioning, ability to get around independently, participation in sports.* Patients' reports about their competence to perform each physical activity (run, swim, ride a bike, walk upstairs, walk downstairs) were coded, so that lower scores indicate more difficulty. Ability to drive was recorded in terms of four categories: (too young = 1, unable to drive but of an appropriate age = 2, able to drive an automatic car only = 3 and able to drive a manual car = 4). Results are shown in Table 2. There was variability in competence on all tasks. Although all but one was able to get up and down stairs, a higher percentage reported difficulties with swimming or riding a bike, and only five (12%) were able to run. In addition to these questions, we asked how many had mobility problems at school or work. No difficulties were reported by 38%. Others had difficulties with steps (43%), or being in a crowd (18%). The problem of crowds was most acute for children at school who often were allowed to leave lessons early in order to avoid the between-lesson crush.

None of the sample reported that they took part in contact sports at least at any competitive level. (Many males qualified this and reported that they did play 'friendlies'.) Participation in all sports was generally very low, with patients commenting that although they were allowed to play non-contact sports, their inability to run very much limited what they could do. Five males and two females expressed considerable regret that they could not participate in sports. Two patients reported difficulties with travelling, due to problems of keeping the leg in one position for any length of time.

Seventeen had taken up some activity to make up for the fact that they could not participate in active sports (e.g. pool, fishing, breeding dogs, sailing). In terms of leisure activities, the majority (66%) did not belong to any clubs. Three (8%) belonged to sedentary clubs (chess), eight (20%) belonged to a sports club and one belonged to a club for the disabled.

Patients were not directly asked whether or not they understood that there was a risk they might have been treated by amputation. However, 23 raised the question of amputation themselves. None felt they had been offered a choice in treatment, rather that prosthesis was the preferred policy of the hospital. All were pleased they had had a prosthesis rather than an amputation. Only one reported having some regrets given the fact that he had not achieved as good mobility as he had hoped. Those who did not discuss the possibility of amputation were younger than those who did ($p < 0.05$).

*(iv) Pain and body image.* Ten (24%) reported that they had no pain associated with the prosthesis at all. Three (7%) reported pain when it was cold, and six (15%) reported pain only after very strenuous activity. Nineteen reported more serious and continuous pain. As a result, six (15%) reported occasional use of analgesics and four reported regular use.

Seven (two males and five females) of the sample were distressed by their scar. The remainder were not embarrassed or at all self-conscious of the scar, and six (four males and two females) reported themselves quite proud ('I wear it like a medal'). Much more distress was experienced in relation to muscle wasting, uneven growth and mis-shapen knees ($n = 25$). Many continued to feel embarrassed by the appearance of their leg; among the males, seven reported that they did not wear shorts or swimming clothes, while among the females, eight always chose to wear trousers or long skirts.

*(v) Well-being.* Three patients were receiving or seeking psychiatric help. In all three cases, extreme fear about recurrence was the major concern.

Patients over 18 years completed the SF-36 ($n = 30$). Mean scores for the group on the separate subscales are shown in Table 3 along with the published norms. A series of $t$-tests to compare mean differences was conducted. The group had scores below population norms on all subscales, and these were significant on physical functioning, physical role performance, pain, general health and social functioning (see Table 3). In addition to the normed scores, patients were asked to rate their health now compared with 1 year previously. Three reported that their current health was 'much better' or 'slightly better' than the previous year, 17 that it was the same, and 2 that it was 'worse' or 'much worse'.

*Fertility.* Among the 18 patients over 16 years of age, two females and one male had children. (There were four children in total.) The remainder were aware that they might be unable to have children.

**Table 3.** *Population norms (n = 173) and mean scores for sample on SF-36 and subscales (n = 30)*

| Mean | Physical function | Role performance | Pain | General health | Vitality | Social function | Emotional | Mental health |
|---|---|---|---|---|---|---|---|---|
| Population | 92 | 89.1 | 80.8 | 76.7 | 62.5 | 83.9 | 83.0 | 74.7 |
| Sample | 67.2 | 75.8 | 65.37 | 67.6 | 59.6 | 74.2 | 79.3 | 70.6 |
| *t* | 6.3** | 2.0* | 3.6** | 2.4* | 0.8 | 2.2* | 0.5 | 0.9 |

**$p < 0.01$, *$p < 0.05$.

*(vi) Functional evaluation rating.*[18] Scores were calculated for 39 patients, and the mean rating was 63% (range = 40–80%). For 16 patients, Enneking scores were also available from previous clinic visits made within 6 months. Ratings made by these 16 patients in their homes were lower than those made by surgeons at the most recent clinic visits (mean at home = 75%; clinic = 69%; $p < 0.05$).

*Evaluation of the interview.* Eighty-three per cent were not at all distressed by the interview, 7% 'a little' and 5% 'very much'.

### Discussion

This study suggests that there is some variability in physical and psychological outcome among young people treated with endoprosthetic replacements. As a group, they appear to do well in public examinations, despite lengthy school absence. While the majority were not distressed or felt able to disguise the appearance of their leg with the right clothes, a proportion went to some lengths to disguise the way they looked.

They all report some limitations on their mobility. This is more of a disadvantage for those at school who are expected to participate in PE programmes compared with those who are at work. Whether at school or work, patients reported being afraid of being knocked, and as a result, tended to avoid crowded places. For some, the lack of a visible handicap represented a disadvantage of the prosthesis over an amputation, since they felt that others were less likely to make allowances. None reported that they experienced restrictions in terms of their opportunities to socialize or travel. None expressed regret about the course of treatment that had been taken.

Our data suggest that, on the standard functional evaluation of reconstructive procedures,[18] patients rate their mobility and satisfaction with the treatment lower than surgeons. Several studies point to parallel findings, that patients or their families rate quality of life to be lower than ratings made by medical staff.[19] Such discrepancies should not be unexpected, and may be attributed to the surgeons' more limited awareness of the patient's functioning in a range of situations, rather than simply in a hospital setting. In addition, patients may very much want to present themselves to medical staff in a positive light. Even so, it is important to recognize that surgeons may inevitably be unaware of the degree to which patients experience difficulties in their daily lives.

From an educational point of view they performed well. Despite very lengthy school absences, many individuals had reached a satisfactory level of education. However, academic progress is certainly vulnerable, as evidenced by the number repeating a school year.

The majority of patients were 'not at all' or 'a little' distressed by the interview. Many patients were, in fact, very positive about their involvement, emphasizing that this was their first opportunity to talk the 'whole thing' through. Although anyone who experienced distress was invited to terminate the interview, no one wished to do so.

The extent to which quality of life is compromised is dependent on how far an individual is prepared or able to develop other skills to compensate for the inevitable lack of physical strength and mobility. In this respect, those individuals with more academic leanings are undoubtedly in better positions than those who would expect to take manual or unskilled work. The limitations experienced by these patients, especially males, may be aggravated in environments where the stereotype of appropriate male work is physical. It is this group of patients (male unskilled) who may need specific counselling to deal with their disability. All recognized that some jobs which involved too much standing or lifting were not possible. These restrictions were of course more limiting for those with less education, who might have expected to work in shops, hairdressing, etc. Others would not consider jobs which involved wearing a uniform which would fail to conceal their scar (e.g. air hostess).

Although the group appears to be functioning as well as might be expected for a group from very different social backgrounds, the majority were still young and in relatively protected environments. Most were in school or higher education and still living with parents. As yet, they have not experienced difficulties in obtaining employment, although given their functional limitations, a proportion at least must expect to experience this. The extensible prosthesis is a temporary device intended to ensure equal limb length throughout childhood and prepare the limb for subsequent insertion of an adult prosthesis. Consequently, it must be anticipated that children will require revision to the adult type at some stage. This will inevitably mean a major disruption in further education and employment prospects. The need for longer-term surveillance of these patients appears, therefore, to be essential.

There is no doubt that limb-salvaging surgery of the kind experienced by these patients is potentially a major set-back to the attainment of a normal life-style. Treatments are very lengthy, the interruptions to normal school or work attendance great, and the possibility of recurrence or problems with the prosthesis are high. The alternative—amputation—does not appear to be associated with less morbidity[20] and may be less acceptable to patients from social and psychological perspectives. Even so, it is not possible to predict the function of a prosthesis when initially embarking on surgery. Our data provide some baseline against which to inform patients and their parents of the likely outcome in terms of physical function. Although it might be useful to have comparable data involving other treatments, there is no obvious control group against which to compare the functioning of these patients. Given that limb saving is the treatment of choice (at least from patient perspectives), it is not appropriate to compare this group with those who have experienced amputation, since at least in our centre, patients are only treated by amputation where limb salvage fails. However, the question of whether or not experience of a prosthesis enables patients to better accept an amputation if it becomes necessary is a very real question for future work.

Future work must, therefore, seek to clarify ways in which quality of life can be optimized for young people undergoing limb salvage procedures. More systematic coordination between national and regional centers at all levels (medical, nursing, social work and physiotherapy) is necessary, as well as the establishment of a counselling service which is informed about the particular needs of these patients.

### Acknowledgement

This work was funded by the Cancer Research Campaign (CP1019/0101).

### References

1 Robertson CM, Hawkins MM, Kingston JE. Late deaths and survival after childhood cancer: implications for cure. *BMJ* 1994; 309:162–6.

2 Finn HA, Simon MA. Limb salvage surgery in the treatment of osteosarcoma in skeletally immature individuals. *Clin Orthop* 1991; 262:108–18.

3 Gebhargt MC, Jaffe KA, Mankin HJ. Bone allografts for tumors and other reconstructions in children. In: Langlais F, Tomeno B, eds. *Limb salvage—major reconstructions in oncologic and nontumoral conditions*. Berlin: Springer, 1991; 561–72.

4 van Nes CP. Rotation-plasty for congenital defects of the femur. *Br J Bone Joint Surg* 1950; 32B:12–16.

5 Kotz R, Salzer M. Rotation-plasty for childhood osteosarcoma of the distal part of the femur. *Am J Bone Joint Surg* 1982; 64A:959–69.

6 Gottsauner-Wolf F, Kotz R, Knahr K, *et al.* Rotation-plasty for limb salvage in the treatment of malignant tumors at the knee. *Am J Bone Joint Surg* 1991; 73A:1365–75.

7 Scales JT, Sneath RS. The extending prosthesis In: Coombs R, Friedlander G, eds. *Bone tumor management*. London: Butterworth, 1987; 168–77.

8 Sneath RS, Carter SR, Grimer RJ. Growing endoprosthetic replacements for malignant tumors. In: Langlais F, Tomeno B, eds. *Limb salvage—major reconstructions in oncologic and nontumoral conditions*. Berlin: Springer, 1991; 573–8.

9 Tupman GS. A study of bone growth in normal children and its relationship to skeletal maturation. *Br J Bone Joint Surg* 1962; 44B:42–67.

10 Cool WP, Grimer RJ, Carter SR, *et al.* The outcome of extensible endoprosthetic replacements of the proximal tibia. *Br J Bone Joint Surg* 1995; 77B Suppl 3:31.

11 Rougraff BT, Simon MA, Kneisl JS, *et al.* Limb salvage compared with amputation for osteosarcoma of the distal end of the femur. *Am J Bone Joint Surg* 1994; 76A:649–56.

12 Weddington WW, Segraves KB, Simon MA. Psychological outcome of extremity sarcoma survivors undergoing amputation or limb salvage. *J Clin Oncol* 1985; 3:1393–9.

13 Sammallahti P, Lehto-Salo P, Maenpa H, *et al.* Psychological defenses of young osteosarcoma survivors. *Psycho-Oncol* 1995; 4:283–7.

14 Eiser C. Choices in measuring quality of life in children with cancer: a comment. *Psycho-Oncol* 1995; 4:121–31.

15 Spieth LE, Harris CV. Assessment of health-related quality of life in children and adolescents: an integratvie approach. *J Pediatr Psychol* 1996; 21:175–94.

16 Jenney MEM, Kane RL, Lurie N. Developing a measure of health outcomes in survivors of childhood cancer: a review of the issues. *Med Pediatr Oncol* 1995; 24:145–53.

17 Jenkinson C, Coulter A, Wright L. Short form 36 (SF 36) health survey questionnaire: normative data for adults of working age. *BMJ* 1993; 306:1437–40.

18 Enneking WF, Dunham W, Gebhardt MC, *et al.* A system for the functional evaluation of reconstructive proceedures after surgical treatment of tumors of the musculoskeletal system. *Clin Orthop Related Res* 1993; 286:241–6.

19 Mulhern RK, Crisco JJ, Camitta BM. Patterns of communication among pediatric patients with leukemia, parents and physicians: prognostic disagreements and misunderstandings. *J Pediatr* 1981; 99:480–3.

20 Simon MA, Ascliman MA, Thomas N, *et al.* Limb-salvage treatment versus amputation for osteosarcoma of the distal end of the femur. *J Bone Joint Surg* 1986; 68A:1331–7.
